# Conflict Behaviour Frequency During Show Jumping Competitions: A Practical Study

**DOI:** 10.3390/ani16111620

**Published:** 2026-05-26

**Authors:** Isabella Torres Nothaft, Felipe Gomes Ferreira Padilha, Giullia Buriti Meriade, Juliana da Silva Leite, Ana Maria Reis Ferreira

**Affiliations:** 1 Programa de Pós-Graduação em Medicina Veterinária (Clínica e Reprodução Animal), Universidade Federal Fluminense, Niteroi 24230-321, RJ, Brazil; felipepadilha@id.uff.br; 2 Faculdade de Medicina Veterinária e Zootecnia, Campus Botucatu, Universidade Estadual de São Paulo, Botucatu 18618-687, SP, Brazil; giulliaburiti@gmail.com; 3 Departamento de Patologia e Clínica Veterinária, Universidade Federal Fluminense, Niteroi 24230-321, RJ, Brazil; jsleite@id.uff.br (J.d.S.L.); ana_ferreira@id.uff.br (A.M.R.F.)

**Keywords:** equestrian sports, sport horses, adverse behaviours, equine welfare, equine stress behaviour

## Abstract

With society’s ever-growing concern for animal welfare, the equestrian industry has focused on passing and enforcing new rules to protect the main athlete, the horse. In jumping, courses go by quickly, with judges using the occurrence of conflict behaviours to assess any welfare breach. This study aimed to evaluate the overall frequency of such behaviours in Brazilian Sport Horses during elite show jumping competitions in Brazil. Conflict behaviour displays were analyzed in 120 different horse–rider pairs in jumping competitions in Brazil. All videos were observed at a slowed-down speed, with the frequency of behaviours being recorded. The observed behaviours included head shaking, tail swishing, neck hyperflexion, excessive pulling on the reins, kicking, bucking, rearing, and disobedience. All horses (100%) presented at least one type of conflict behaviour, with head shaking (100%, *n* = 120, IQR 3–7, range 1–18) being the most common one (*p* < 0.001) and tail swishing (45.83%, *n* = 55, IQR 1–10, range 1–29) being the second most common (*p* < 0.001). Most horses showed only one (41.67%, *n* = 50) or two (43.33%, *n* = 52) different types of behaviours, with few episodes of each throughout the course. Those findings were in line with studies in other disciplines, as the competition environment offers a series of challenging and stressful situations. The low levels of conflict observed in most horses indicate that the current horse welfare rules are working and must continue to be reinforced to consistently protect the horses.

## Simple Summary

In recent years, several new rules focused on the welfare of horses and fair play in show jumping competitions have been approved. Since courses last only about 1 min, judges must have a strong understanding of horsemanship dynamics to quickly assess any welfare breach. Conflict behaviours, which indicate discomfort, confusion, resistance, or hyperreactivity to the rider’s aids, are a typical sign of difficulty in managing the physical or mental load and are often considered a possible welfare breach. Based on observations of 120 show-jumping courses, this study aimed to evaluate the overall frequency of these behaviours in Brazilian Sport Horses during elite show-jumping competitions in Brazil. It was observed that all horses presented at least some sort of conflict behaviour, with head shaking being the most common one, followed by tail swishing, mostly with a few episodes throughout the course. Those findings were expected, as the competition environment offers a series of challenging and stressful situations, especially in high-level equestrian sports. The low levels of conflict observed in most horses indicate that the current horse welfare rules are working and must continue to be reinforced to consistently protect the horses.

## 1. Introduction

The concept of a ‘social license to operate’ (SLO) has finally entered the equestrian world, raising concerns among various stakeholders in the sporting industry [1]. A social license is said to exist when an activity has the acceptance of society, allowing it to operate [2]. Recent scandals in the riding world, such as the suspension of major athletes right before the 2024 Olympic Games, have cast a negative light on the sport, leading to low public acceptance [3]. To increase society’s trust in equestrian sports, regaining the credibility lost over the years, the equestrian athletes and stakeholders must act, being transparent in their actions and bringing to light the values of horse welfare, avoiding the tendency riders have to normalize coercive corrections and overlook the causes of undesired behaviours [4,5]. This includes new legislation, new ethics-based practices, proactive control, and optimization of equine welfare across all competitions [6].

Aiming to reduce the occurrence of negative behaviours and ensure equine welfare, both the International Federation for Equestrian Sports (FEI) and the Brazilian Equestrian Sports Confederation (CBH) have developed and applied several rules to prevent inappropriate and harmful training techniques [7,8]. In a recent document issued in 2022, FEI has stated that “horses must only undergo training that matches their physical capabilities and level of maturity for their respective disciplines. They must not be subjected to methods which are abusive or cause fear.” Since the 2024 FEI’s Jumping Rulebook (article 241.4), an important new welfare rule was also added, in which the president of the ground jury may ring the bell to eliminate an athlete/horse combination while a round is ongoing if this action would be in the best interest of the well-being and safety of the horse. With the creation and enforcement of those rules, there will be fewer instances of conflict stemming from incorrect training methods [9].

However, before intervening, the FEI judge must determine which actions by the rider and reactions by the horse constitute negative factors that place the welfare of the horse–rider duo at risk. First of all, conflict behaviours (CBs) can be defined as behaviours seen in the ridden horse that indicate stress, discomfort, confusion, resistance, or hyperreactivity to the rider’s aids [10,11]. CBs are normally exhibited by animals with difficulties in dealing with the physical or mental load asked of them, and these are rarely reported in wild horses or those in group pastures [12].

Currently, the FEI has adopted a guideline developed by the German Equestrian Federation to help its officials and other stakeholders recognize negative behaviours and know when to intervene. The horse’s behaviour must always be monitored closely, so that the observer may evaluate which path the rider is taking. Is this horse-friendly behaviour? If so, no action is needed. Is the behaviour non-horse-friendly? Immediate action must be taken to ensure the horse’s welfare. But now comes the grey area, into which most behaviours fall, and in which most caution is needed: conspicuous conduct. From there, three paths may be taken. The first one, in which the behaviour improves and becomes friendly again, is the most sought after. The horses relax, stop exhibiting signs of stress or conflict behaviours, and return to work pleasantly, indicating a return to equilibrium in which horse welfare principles are being met. The opposite route, in which the behaviour becomes unfriendly, must always be avoided. In this case, indicators of stress on the horse increase, with more signs of stress and anxiety, often reflected in increased conflict behaviours. Riders may also be endangering the horse’s well-being by using excessive whips, spurs, rein aids, or placing the horse in a risky situation, and thus must be stopped immediately. The third route is when the observer deems the behaviour still acceptable under the circumstances and continues to wait and observe the outcome of that interaction [13]. These include a single light use of whips or spurs to guide the horse, a moment of hyperflexion as the horse adjusts to rein aids, or even stronger vocal cues when a horse spooks. It embodies a single or short, doubtful attitude from the rider towards the horse, which should fall back into a horse-friendly manner. The evaluation of this route depends almost entirely on the observer’s knowledge of horsemanship, whether a veterinarian, judge, steward, groom, or any other stakeholder. It must always be kept in mind that, in case of doubts about the safety or acceptability of a certain practice, it should be stopped until it can be proven not to cause harm to the horse [14].

The main goal of equestrian athletes is to achieve conflict-free cooperation with their horses to improve rideability, but this is not always possible [15]. Therefore, horse-friendly solutions must be used to address any issues that may arise, avoiding negative or non-horse-friendly actions. The way of riding must be harmonious, with the correct application of aids, in a way that is comprehensible and fair for the horse, allowing for supple and well-balanced gaits. The horse should be on the bit, with its mouth mostly closed, salivation on the lips, and the face close to the vertical position with no artificial restriction of the horse’s head position [16]. During certain movements or at specific moments, the horse may go behind the vertical or open its mouth as a form of resistance. Still, those moments must be corrected promptly to return to a horse-friendly riding style. The position of the eyes and ears, as well as the nostrils and breathing patterns, may also help to recognize a relaxed working horse from a tense or scared horse. They should remain attentive, focusing on the surroundings and the rider, without appearing laid-back, with bulging eyes, or raised nostrils. One other body signal the horse gives the rider and the observers is tail swishing. The tail should be carried lightly, swinging freely with the gait’s movement and being swished to ward off flies and other insects. Constant tail swishing or a tail carried pinched up for a prolonged period could indicate discomfort and should be addressed. More extreme reactive behaviours, such as bucking, rearing, and kicking, or refusals in showjumping competitions, should also be closely looked at, as a means to evaluate if it is just a joyful expenditure of energy or if it actually indicates a physical or behavioural problem [10,13,17,18,19,20].

While most conflict behaviour studies focus on dressage and jumping [18,20,21], some work has been conducted in other disciplines, such as eventing horses [22], Icelandic gaited horses [23], and racing trotters [24]. The more different equestrian disciplines are studied, the more conflict-behaviour triggers can be understood and avoided for all horses.

Certain studies, such as the one conducted by Fiedler et al. [24], indicate that officials, veterinarians, and participants should be responsible for implementing management practices and ensuring the horse’s well-being. Specifically, in the FEI-regulated jumping competitions, the horses must be overlooked and accompanied by the steward whenever they are being trained or warmed up in an event, as a way to guarantee the fair play of the competition and the horse’s well-being. Since the stewards are always in close contact with the horse–rider pairs and chaperone them for extended riding periods, it becomes clear when a rider is riding in a horse-friendly manner or when they use excessive, unfriendly commands. However, for the ground jury, which accompanies each pair for roughly 90 s during show-jumping competitions, observing and acting might not be as straightforward. The horse needs to be seen in its entirety, not just one specific criterion or behaviour used to deem a breach of equestrian welfare, thereby leading to the elimination of the pair.

In this manner, even with the approval of new rules and regulations that ensure the welfare of the horse and fair play in the competition, judges sometimes lack the necessary tools or understanding of horsemanship dynamics to assess the situation during the few seconds of a competition round. To better understand the conflict behaviours expressed in the competition arena and to help establish guidelines for the ground jury, behavioural studies are needed. Thus, this study aims to observe the overall frequency of conflict behaviour in Brazilian Sport Horses during elite show jumping competitions in Brazil.

## 2. Materials and Methods

This study has been approved by the Ethics Committee in Animal Use (CEUA) of the Fluminense Federal University under protocol number 6181221021/2022.

### 2.1. Study Sample

Conflict behaviour displays were analyzed in 120 horse–rider pairs competing in high-level show jumping competitions in Brazil, all of whom participated in certified National Jumping Competitions from 2022 to 2024. All horses selected for this study were purebred Brazilian Sport Horses (BH), registered with the Brazilian Association of Equestrian Horse Breeders (ABCCH). Horses from other breeds, even if imported to Brazil and registered in the BH studbook, were excluded from this study. The horse–rider pairs represented Brazil and were registered through the following state federations: Rio de Janeiro, São Paulo, Minas Gerais, Brasilia, Paraná, and Rio Grande do Sul. Before the beginning of each course, several different pieces of information were recorded: the rider’s jumping category, as well as the horse’s breed, year of birth, and sex (Table 1). The results of each pair were recorded, with time penalties and jumping faults specified, always using FEI-approved timing systems. Six of the horse–rider pairs received no final result for their competition course due to either elimination or retirement. The data collected was anonymized.

In this study, all courses analyzed were held in official National competitions that strictly followed the FEI and CBH rules. All horses were vetted and approved, meaning they were fit to compete. Additionally, they were always under the scrutiny of FEI veterinarians, judges, and stewards in the stables, training areas, warm-up, and competition arenas, meaning any horse found lame or with any welfare issue was excluded from competition. Since all competitions happened under the FEI and CBH rules, all materials used in training and competition were checked by stewards to guarantee that they were allowed and did not cause any harm to the horse.

The competition heights ranged from 1.20 m to 1.40 m, with both professional and non-professional riders competing side by side. The grouping levels used followed the Brazilian system [25], taking into account each rider’s age and jumping height (Table 2).

### 2.2. Data Collection

All courses were analyzed from video footage available on the HorsePix group’s YouTube channel [26]. Since only publicly available videos were used, there was no need for ethical or image-use approval to comply with national legislation. All online recordings were viewed at a slow speed (×0.25), enabling a detailed evaluation of the horse’s behaviour during the entire course. A single observer, who is a veterinarian, an FEI steward, and a national jumping judge, analyzed all video recordings using a methodology similar to that of Jastrzębska et al. (2017) [11] and Górecka-Bruzda et al. (2014) [17]. The use of a single observer resembles actual jumping competitions, in which the President of the Ground Jury (or another jury member selected by the President) is responsible for evaluating equine welfare and, if needed, stopping the pair during the course for welfare reasons [8].

Regarding conflict behaviours, an ethogram (Table 3) was applied to analyze the following behaviours: head shaking, tail swishing, neck hyperflexion, excessive pulling on the reins, kicking, bucking, rearing, and disobedience. Each instance of one of those behaviours, regardless of its cause in the competition arena, was recorded in a specific ethogram control spreadsheet. Those behaviours were chosen because they are more discernible and easier to see during the fast-paced riding competition, in which certain behaviours, such as opening the mouth or flaring the nostrils, might not be seen by the competition judge during the course [27].

The occurrence of each behaviour during the course was recorded. Descriptive statistical analysis was performed on all data, including frequencies, means, medians, and ranges. The quartile values, IQR, and outlier points were also calculated. To assess whether there was a significant difference in the frequencies of the conflict behaviours, the data were checked for normality using the Shapiro–Wilk test. After establishing a non-normal distribution, the Related-Samples Friedman Test was applied to evaluate differences in the occurrence of each behaviour, with the Wilcoxon signed-rank test with Bonferroni correction applied post hoc. The Kruskal–Wallis Test was used to compare each behaviour by horse sex, with Dunn’s post hoc test. All statistical analyses were performed using IBM SPSS Statistics (Chicago, IL, USA), version 32, and complete results can be found in the Supplementary Materials.

## 3. Results

In total, 120 different Brazilian Sport Horses were observed in this study. The two oldest horses were born in 2000 and were 22 and 23 years old at the time of observation, while the youngest horse was born in 2016 and had just turned 6 at the time of analysis. The average year of birth was 2009.28 ± 2.73, with the mean being 2010. Regarding sex, 55% of the horses were males (*n* = 66), and the other 45% were females (*n* = 54).

Regarding the riders, they were sorted by Brazilian riding level, taking into account both the rider’s age and competition level. Of all athletes, 65 (54%) were professionals competing in the Senior category. The remaining 55 riders (46%) were non-professionals, divided into the following categories: 12 in the Amateurs, 9 in the High Amateurs, 4 in the Young Riders, 6 in the High Young Riders, 2 in the Juniors, 2 in the Children, 6 in the Pre-Juniors, and 14 in the Under 25.

When analyzing the normality of conflict behaviour occurrence, the Shapiro–Wilk test indicated that none of the behaviours were normally distributed (*p* < 0.001), with skewness > 1 and kurtosis > 2. The visual inspection of both the Q-Q plot and histogram graphs confirmed this finding. Regarding the influence of sex on the occurrence of conflict behaviours, there was no statistically significant difference (*p* > 0.05) for 7 of the 8 behaviours analyzed. The only behaviour that showed a significant difference was disobedience (*p* = 0.027), with females exhibiting more disobedience.

**Figure 1:**
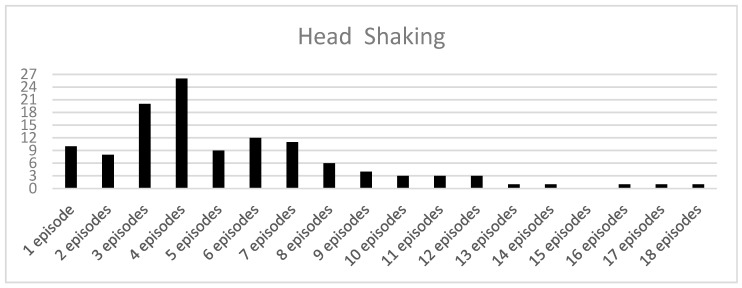
Number of horses per number of head shaking episodes during show jumping courses.

The most observed conflict behaviour was head shaking. All horses analyzed (100%) presented head shaking during the course, with at least one episode observed. The average number of episodes per jumping course was 5.38 ± 3.44, with a median of 4 episodes, an IQR of 3–7 episodes, and a range of 1–18 episodes. A total of 10 horses displayed only 1 episode of headshaking. In contrast, the maximum number of headshakes in a single course, 18 episodes, was exhibited by a 2009 horse with a senior rider (Figure 1). There was a significant difference (*p* < 0.001) between head shaking and all other behaviours analyzed.

The second most common conflict behaviour was tail swishing. This behaviour also differed significantly (*p* < 0.001) from all other behaviours selected for this study. It was observed in 55 of the 120 horses (45.83%). When analyzing the horses that exhibited this behaviour, an average of 6.92 ± 7.37 episodes was observed, with a median of 3 and an IQR of 1–10. It ranged from 1 to 29 episodes per course, with the greatest amount of tail swishing being displayed by a 2011 mare with a senior rider.

**Figure 2:**
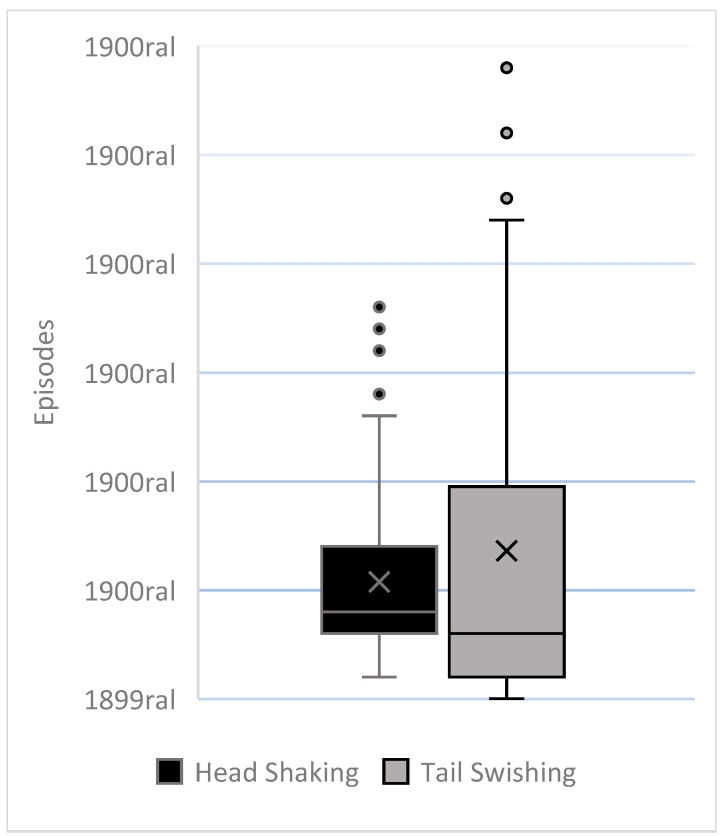
Box-plot graph representing the distribution of conflict behaviour occurrence for head shaking (black) and tail swishing (grey). The dots above indicate outliers, while the X marks the mean value.

**Figure 3:**
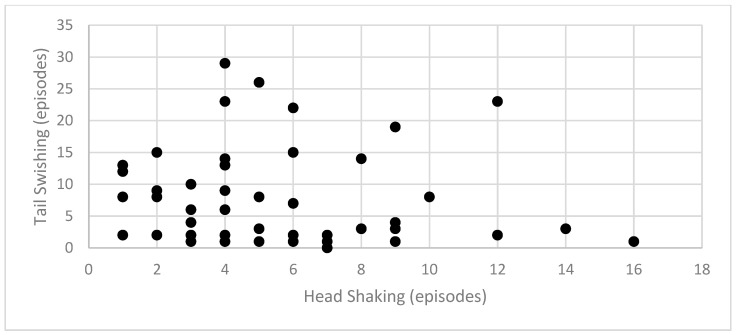
Distribution of conflict behaviours in horses: both head shaking and tail swishing were observed during the course.

When looking at the two most common behaviours, they showed a similar distribution, with most horses having few episodes and only a few outliers with more episodes (Figure 2), even though their frequencies differed statistically (*p* < 0.001). Head shaking had a smaller standard deviation, resulting in more compact results, compared to tail swishing, which stretched the numbers. When analyzing horses that exhibited both behaviours, it can be observed that most horses with a high level of one behaviour had a low level of the other, and vice versa (Figure 3).

The following behaviours were all considered statistically similar in frequency (*p* > 0.05) and occurred in 10% or less of the courses analyzed. The conflict behaviour of neck hyperflexion was counted when it lasted more than 3 s during the course. Only 11 of the 120 horses (9.16%) displayed this behaviour, with an average of 1.63 ± 0.92 episodes per horse. A single episode was observed in 6 of them; 4 of the horses had 2 episodes; and the horse with the greatest number of episodes was a 2011 horse with a senior rider, with 4 distinct episodes of hyperflexion.

The pulling of the reins occurred in only 6 of the 120 horses (5.00%), having an average of 1.50 ± 0.84 amongst them. It happened 1 to 3 times in each course. In total, 4 horses had only 1 episode of pulling on the reins, while a 2011 mare with a senior rider had 2 episodes, and a 2005 mare had 3 episodes, the most recorded in this study. The mare with 2 episodes also had 4 episodes of head shaking, 29 episodes of tail swishing, and one refusal, while the mare with 3 episodes also had 12 episodes of head shaking. None of the horses that presented with pulling of the reins presented with hyperflexion of the neck, but all of them also showed tailed swishing.

The conflict behaviours considered more extreme expressions of discontent by the horse, such as bucking, kicking, rearing, and disobedience, occurred very infrequently in the courses analyzed. Bucking occurred in only 2 horses (1.66%): one horse, a 2011 horse with a senior rider, had a single episode; the other, a 2004 grey horse with a senior rider, had 2 episodes. The only other conflict behaviour both horses showed was head shaking, with 5 episodes each, respectively. The kicking behaviour occurred in only one of the 120 horses observed (0.83%), a 2007 male horse ridden by a senior rider. This horse had 7 episodes of kicking, 4 episodes of head shaking, 9 episodes of tail swishing, and 1 episode of rearing.

Rearing was also a rare behaviour, occurring in only 3 of the 120 horses (2.50%), with each horse having a single episode. The first horse was the 2007 male horse mentioned above. The second was a 2008 mare ridden by an amateur rider, who also presented with 7 episodes of head shaking and a refusal, after which the rearing behaviour occurred. The last horse was a 2011 horse ridden by a senior rider, who also exhibited 4 moments of head shaking during its course.

The last observed conflict was disobedience. All observed disobedience was refusal or resistance, occurring in 12 of the 120 horses (10%), with only 1 per course in 11 of those horses. The twelfth one, a 2007 mare ridden by a young rider, had two refusals on the last jump and was thus eliminated. Before the elimination, the mare also exhibited 14 counts of head shaking and 3 of tail swishing. Of those 12 horses with refusals, only 4 were ridden by Senior riders. The remaining horses were ridden by one Under 25 rider, one High Young Rider, one Young Rider, 2 Amateurs, 2 Pre-Juniors, and one Junior rider.

**Figure 4:**
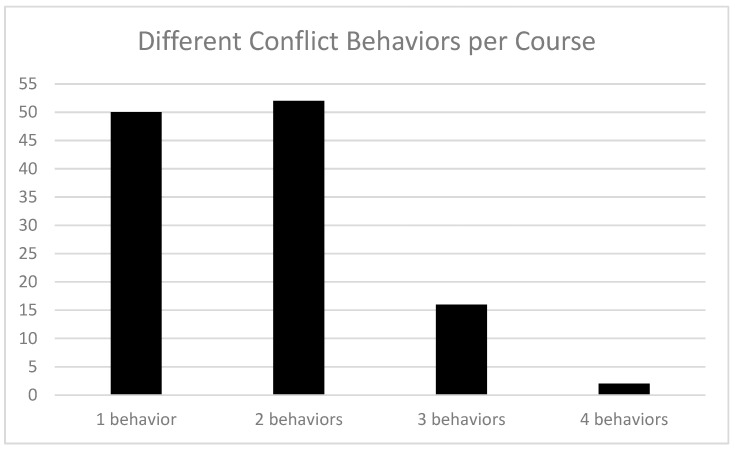
Number of horses per number of different conflict behaviours per show jumping courses.

When looking at the overall amount of conflict behaviours during the jumping course, all horses showed between 1 and 4 different conflict behaviours (Figure 4). There were 50 horses with only one type of CB: head shaking. In total, 52 of the analyzed horses showed 2 types of conflict behaviour: 38 combined head shaking with tail swishing; 6 with hyperflexion of the poll; 5 with disobedience; 2 with bucking; and 1 with rearing. Of the 16 horses with 3 different types of conflict behaviours, 15 of them had both head shaking and tail swishing, combined with 5 cases of hyperflexion of the neck, 5 of pulling on the reins, and 5 of disobedience. The 16th horse presented with head shaking, rearing, and disobedience. Of the horses with 4 distinct behaviours, the first one was a 2007 gelding ridden by a senior rider, with head shaking, tail swishing, rearing, and kicking. The second horse was a 2011 mare, ridden by a senior rider, exhibiting head shaking, tail swishing, pulling on the reins, and disobedience.

## 4. Discussion

Equestrian show jumping competitions are very fast-paced, with the ground jury evaluating the horse–rider pair for only about a minute per course. This way, by knowing the expected frequencies of certain conflict behaviours during competitions, judges will be able to quickly identify excessive behaviours and act to ensure equine welfare.

In this study, head-shaking behaviour was observed in all courses analyzed and was considered statistically more frequent than the other behaviours (*p* < 0.001). This finding was expected, as the competitive environment presents a series of challenging and stressful situations, especially in high-level equestrian sports [16,17]. A study by Hamilton et al. [19] found similar results, with conflict behaviours in over 97% of the dressage movements analyzed. As horses are extremely sensitive beings, it is natural that they show some reaction during the competition, even with a slight shake of the head in response to the rider’s aid, aiming to adjust the distance or make a sharp turn to face the next obstacle. Authors such as Murphy et al. [29] and Telatin [30] claim that horses might display a headshaking behaviour close to the jumping obstacles to be able to view them and correct their approach to them, while others [31] propose that this sort of conflict behaviour is directly related to the rider’s aid for reducing speed.

On average, horses had 5.38 ± 3.44 episodes, with the majority of horses having seven or fewer episodes of head shaking per course. Those findings indicate that although all horses had at least one occurrence of conflict behaviour, these occurred at low frequencies. When there are only a few occurrences in a course, it usually indicates a single moment of adjustment, in which the horse clashes with the rider’s aid and reacts by shaking its head. This allows the judge to evaluate whether a certain negative behaviour evolves into an unfriendly riding style or returns to a horse-friendly riding style [13].

The second most common CB observed in this study was tail swishing, which was considered an intermediate-frequency behaviour (*p* < 0.001). This finding is similar to those reported by Górecka-Bruzda et al. [17], who also described head reactions and tail swishing as the most prevalent CBs in show jumping competitions in Europe. Dyson and Pollard [20] recorded tail swishing in 29% of the dressage horses analyzed, while Christensen et al. [22] recorded a 58.3% frequency of tail swishing in Icelandic horses. In this study, this behaviour was present in over 40% of the courses, with a wide range of 1 to 29 episodes per course, indicating that this reaction is highly individual to each horse. The mare, which exhibited 29 episodes throughout her course, is described by her rider and breeder as very reactive and tense, both when ridden and on the ground, and is known for her difficult temperament. The other episodes were demonstrated mostly when the rider needed greater control, strength, and collection of the horse, such as in short lines or difficult obstacle approaches, where the horses had to use considerable strength to overcome the challenges.

A recent study by Fialova et al. [16] corroborates this, as dressage horses at the beginner level, where horse collection is not required, showed a tail-swishing frequency of less than 5% in competitions. Similarly, when analyzing dressage horses, Christensen et al. [18] found that tail swishing was the most common CB, observed in all tests viewed, with 1 to 34 episodes per test. Since dressage requires significant strength and collection from the horse, it supports the present study’s findings as additional evidence of tail swishing under greater demands on the horse.

The behaviours of neck hyperflexion, pulling on the reins, disobedience, rearing, bucking, and kicking all occurred at low frequencies across the analyzed courses (*p* > 0.05). Hyperflexion of the neck was the third most common behaviour observed in this study, occurring in about 9% of the courses viewed. It occurred at low frequency, with only one to four episodes per course observed. This number is much lower than those seen in dressage competitions [17,19], since in show jumping, horses are usually asked for speed and power, unlike the high collection demanded in dressage. The low frequency with which this CB was observed in show jumping is a good indicator, suggesting that few horses are subjected to this detrimental position in this particular equestrian sport in Brazil.

Similarly, as observed in dressage competitions [17,19], excessive hand aid could be the main cause of pulling-on-the-reins behaviour. This particular conflict behaviour was observed in only 5.1% of courses, with a higher frequency in mares. Just as in the hyperflexion of the neck occurrences, most of the horses used flash bridles, which prevent the horse from opening the mouth and avoiding the pressure of the bit, thus giving the horse the only option of avoiding the contact by pulling down the reins. This behaviour usually throws off the rider’s balance, which in turn causes slack on the rein and a reduction in the pressure in the mouth. When this does not happen, the continued pulling of the reins by the rider may lead to hyperflexion behaviour, which explains why most horses presenting with CB of pulling the reins also show moments of poll hyperflexion.

Bucking occurred in approximately 5% of the courses, with only male horses exhibiting this CB. In a similar manner to the tail swishing behaviour, bucking may happen to express the horse’s discomfort with a particular situation it is being asked to face or as a manifestation of pain, particularly in the back, since the horse tries to remove the rider from its back and reduce the load carried. All of the horses that displayed this conflict wore double-shell hind boots that might have been improperly adjusted, causing them to buck from discomfort or simply because they were not used to wearing them and the pressure they exerted.

The kicking behaviour was observed only in a gelding wearing the double-shell boots, accounting for only 1% of the courses viewed. This particular horse was very reactive, exhibiting several different conflict behaviours during the course. Once again, the use of the double-shell boots might have caused this behaviour. It is important to note that stewards were present at all the competitions analyzed, ensuring that all materials used, including the boots, complied with both the CBH and FEI equipment rules. It is also important to rule out pain as the cause of those behaviours, as horses tend to mask it and display it as aggression [32,33].

Another conflict with very low frequency was rearing, present in only 2% of the courses. All horses that displayed this CB were stallions, which reared before the beginning of the course. Since stallions present greater testosterone levels than geldings, they tend to be more strong-willed and reactive to external sensory inputs, such as the scent left by previous horses. This behaviour did not affect the course or the results, as the horse–rider duos committed no penalties.

The CBs that led to the worst results in the competitions were those in which the horses showed disobedience. In each instance, the horse–rider duo incurred both course and time penalties or was eliminated. Fence refusals occurred in 2% of courses, always by geldings. In contrast, resistances, with the refusal to move in the required direction, happened in approximately 4% of the courses, with both female and male horses. Usually, both behaviours occur in response to an ineffective rider’s aid or to a wrong jumping approach, leaving the horse unable or unwilling to follow the commands. Incorrect use of leg and rein aids, ineffective use of the outer rein, and a bad jumping distance are the most common rider errors that lead to refusals and the penalties that follow. Females were statistically more likely to commit disobedience (*p* = 0.027), but the causes of this difference warrant further study. Hormonal cycles, hormonal inhibitors, or temperament could be among the causes worth investigating.

During FEI-regulated competitions, all of the behaviours mentioned above may occur during the jumping course. They can be directly attributed to stress, incorrect use of the rider’s aids, pain, discomfort from certain materials, environmental factors, or many other causes, but regardless of the cause, they do occur in the competition setting. Even though all horses presented with at least one conflict behaviour, those moments occur in most pairs only a few times per course. This low frequency observed across most behaviours indicates that horses are generally comfortable with the competition environment and its associated stress-inducing factors [20].

For the ground jury, which accompanies each pair for just a few seconds, observing and taking action might not be as easy [27], since the horse needs to be seen in the whole context, and not only in one specific criterion or behaviour may be used to deem a breach in equestrian welfare. The horse–rider duo’s behaviour must be closely monitored at all times and categorized as horse-friendly, non-horse-friendly, or conspicuous [13]. Knowing which behaviour fits can help judges make faster, better decisions, serving as a guideline to follow during the brief time they are in contact with the pair. A future possibility is to combine judges’ evaluations with Artificial Intelligence tools to assess equid behaviour, physical wellbeing, and comfort when ridden [34].

To improve understanding of equine conflict behaviours in competition settings, further research is needed. The present study worked with a small sample size, with only a single course observation per horse–rider pair. Studies with larger sample sizes, focusing on several courses per horse over a jumping season and including different riders, can shed light on the rider’s influence during competition. Additionally, studies that physically examine the horse for signs of pain or discomfort help address one of this study’s limitations, namely, that the cause of the behaviour cannot be determined.

The evaluation of equine behaviour must not be limited exclusively to competition courses; it must be monitored by officials in training arenas and by riders at home at their training centres [3]. When the horse’s well-being becomes a priority across all aspects of their preparation and daily life, the equine community will begin to regain society’s trust and its social license to operate. One major concern is avoiding normalizing the occurrence of conflict behaviours. Even though all horses showed at least some episode of conflict behaviour, the goal must be to achieve rounds without those behaviours. For judges, knowing that low levels of tail swishing and head shaking are common will help prompt action when higher levels are observed, steering the pair toward a horse-friendly approach. Through ethical practices and regulations, alongside proactive control by riders and officials, the show jumping community should gradually improve equine welfare, regaining society’s trust and support.

## 5. Conclusions

This study demonstrates that conflict behaviours occur during show-jumping competitions, with all horses exhibiting at least one episode throughout the course. The most common behaviour was head shaking, present in all of the analyzed courses, followed by tail swishing. Behaviours such as pulling on the reins, bucking, kicking, rearing, and disobedience occurred at very low frequency, occurring in under 10% of courses. These results indicate that while conflict behaviours can be observed during competitions, a moment of higher demand for the horse, they occur infrequently in most cases, allowing judges to visualize them and act upon them when needed to maintain equine welfare. The low levels of CB found in most horses indicate that the current horse welfare rules are working and must continue to be reinforced to consistently protect horses and ensure they are well cared for and prepared for high-level competition tasks. Further research into a larger sample is needed to determine the frequency and, possibly, to establish guidelines to help judges and officials know when to take action during non-horse-friendly, high-conflict behaviour episodes and when only attention is needed during conspicuous behaviour moments. Studies are also needed to clarify how the frequency of conflict behaviours, the horse’s sex, equipment, training, and the rider’s level affect each other.

## Figures and Tables

**Table 1:** Number of horse–rider pairs; age, breed, and sex of the horses; rider categories in the sample (N/A—not applicable).

Parameter	Characteristics	(*n*)	%
Horse–rider pairs		120	100
Eliminated or retired		6	5
Age (±SD) (years)		11.52 ± 2.37	N/A
Breed	Brazilian Sport Horse	120	100
Sex	Male	66	55
	Female	54	45
Rider Categories	Professionals	65	54
	Non-Professionals	55	46
Amongst non-professional riders	Amateurs	21	38.18
	Young Riders	10	18.18
	Youth Classes	24	43.64

**Table 2:** Show jumping grouping levels in the Brazilian system, divided by profession, age, and height of competition [25].

Type of Rider	Division	Category	Age Group (y)	Competitions
Professional	Senior	Senior	Starts at 18	Up to 1.60 m classes
Non Professionals	Amateurs	High Amateurs	Starts at 25	1.30 m classes
		Amateurs	Starts at 25	1.20 m classes
	Young Riders	High Young Riders	15 to 24	1.30 m classes
		Young Riders	13 to 24	1.20 m classes
	Youth Classes	Under 25	16 to 25	1.40 to 1.60 m classes
		Junior	14 to 18	1.40 m classes
		Pre-Junior	14 to 16	1.30 m classes
		Children	12 to 14	1.20 m classes

**Table 3:** Ethogram with a description of the observed conflict behaviours [8,17,22,28].

Behaviour	Description
Head Shaking	The horse shakes its head laterally, up and down, in circles, or tosses, producing a clear movement from the position requested by the rider.
Tail Swishing	Lateral, circular, or up and down motion of the tail that interrupts the natural biomechanical waving motion of the tail during the canter and jumping motions.
Hyperflexion of the Neck	Movement in which the neck is flexed dorsoventrally towards the chest of the horse, positioning the head or nasal plane behind the vertical line, for an extended period.
Pulling on the reins	Quick extension of the neck forward and usually downwards, trying to lengthen the rein contact of the rider.
Disobediences	Includes refusals—when a horse stops in front of an obstacle that it must jump, knocking it down or not (art 246.2.1); run-outs when the horse escapes the control of the rider and avoids an obstacle (art 246.3.1); and resistances—any instance where the horse refuses to go forward, stops for any reason, moves backwards, or makes one or several turns or half turns [8].
Kicking	Movement in which the horse extends one or both of its hind limbs backwards and upwards, usually associated with pinned ears.
Bucking	Movement in which the horse lowers its head while rounding its back and jumps into the air, extending or not its hind legs into the air.
Rearing	Movement in which the horse raises its front limbs from the ground, remaining balanced only in its hind limbs while doing so.

## Data Availability

The data presented in this study are openly available in Mendeley Data at https://data.mendeley.com/datasets/tk8d8gmrdt/1. The videos of the competitions analyzed may be found on the HorsePix YouTube channel at https://horsepix.com.br/salto/ (accessed on 22 May 2026).

## Supplementary Materials

The following supporting information can be downloaded at: https://www.mdpi.com/article/10.3390/ani16111620/s1. The data file attached contains the results yielded by IBM SPSS (v32) for the statistical analysis of this work, including the Shapiro-Wilk test results (Table S1), the Kruskal-Wallis Test with Dunn’s post hoc test results (Table S2), the Related Samples Friedman’s Two-Way Test, with Wilcoxon signed-rank test with Bonferroni correction applied post hoc results (Table S3), and the pairwise graphical comparison (Figure S1).

## Abbreviations

The following abbreviations are used in this manuscript:

SLO	Social License to Operate
FEI	Federation Equestre Internationale
CBH	Confederação Brasileira de Hipismo
CB	Conflict Behaviour
CEUA	Comissão de Etica de Uso de Animais
SD	Standard Deviation
FEI JR	Federation Equestre Internationale Jumping Rulebook
IQR	Interquartile Range
*n*	number
SPSS	Statistical Package for the Social Sciences
