# Supplementary material for: Conflict Behaviour Frequency During Show Jumping Competitions: A Practical Study
Source: Animals (Basel). 2026 May 26;16(11):1620. doi: 10.3390/ani16111620 (PMC13255590; doi:10.3390/ani16111620)

Conflict Behaviour Frequency during Show Jumping Competitions: A Practical Study

Supplementary Material

Table S1: Results of data normality through the Shapiro-Wilk Test

	Kolmogorov-Smirnov[a]			Shapiro-Wilk		
	Statistic	df	Sig.	Statistic	df	Sig.
Head Shaking Behaviuor	,189	120	<,001	,884	120	<,001
Tail Swishing Behaviour	,327	120	<,001	,595	120	<,001
Hyperflexion of the Neck Behaviour	,517	120	<,001	,307	120	<,001
Pulling on the Reins Behaviour	,530	120	<,001	,209	120	<,001
Desobediences	,526	120	<,001	,349	120	<,001
Kicking Behaviour	,528	120	<,001	,065	120	<,001
Bucking Behaviour	,532	120	<,001	,104	120	<,001
Rearing Behavior	,535	120	<,001	,108	120	<,001

a. Lilliefors Significance Correction

Table S2: Tests for comparison between behaviour and horse's sex using the results yielded by the Kruskal-Wallis Test with Dunn's post hoc test.

Hypothesis Test Summary				
	Null Hypothesis	Test	Sig.[a,b]	Decision
1	The distribution of Tail Swishing Behaviour is the same across categories of Sex of Horses.	Independent-Samples Kruskal-Wallis Test	,057	Retain the null hypothesis.
2	The distribution of Hyperflexion of the Neck Behaviour is the same across categories of Sex of Horses.	Independent-Samples Kruskal-Wallis Test	,208	Retain the null hypothesis.
3	The distribution of Pulling on the Reins Behaviour is the same across categories of Sex of Horses.	Independent-Samples Kruskal-Wallis Test	,769	Retain the null hypothesis.
4	The distribution of Desobediences is the same across categories of Sex of Horses.	Independent-Samples Kruskal-Wallis Test	,027	Reject the null hypothesis.
5	The distribution of Kicking Behaviour is the same across categories of Sex of Horses.	Independent-Samples Kruskal-Wallis Test	,366	Retain the null hypothesis.
6	The distribution of Bucking Behaviour is the same across categories of Sex of Horses.	Independent-Samples Kruskal-Wallis Test	,199	Retain the null hypothesis.
7	The distribution of Rearing Behavior is the same across categories of Sex of Horses.	Independent-Samples Kruskal-Wallis Test	,886	Retain the null hypothesis.
8	The distribution of Head Shaking Behaviuor is the same across categories of Sex of Horses.	Independent-Samples Kruskal-Wallis Test	,335	Retain the null hypothesis.

a. The significance level is ,050.

b. Asymptotic significance is displayed.

Table S3a: Tests for comparison between behaviour's frequencies, using the results yielded by the Related Samples Friedman's Two-Way Test.

Hypothesis Test Summary				
	Null Hypothesis	Test	Sig.[a,b]	Decision
1	The distributions of Head Shaking Behaviuor, Tail Swishing Behaviour, Hyperflexion of the Neck Behaviour, Pulling on the Reins Behaviour, Desobediences, Kicking Behaviour, Bucking Behaviour and Rearing Behavior are the same.	Related-Samples Friedman's Two-Way Analysis of Variance by Ranks	<,001	Reject the null hypothesis.

a. The significance level is ,050.

b. Asymptotic significance is displayed.

Table S3b: Tests for comparison between behaviour's frequencies, using the results yielded by the Related Samples Friedman's Two-Way Test, with Wilcoxon signed-rank test with Bonferroni correction applied post hoc.

Pairwise Comparisons

Sample 1-Sample 2	Test Statistic	Std. Error	Std. Test Statistic	Sig.	Adj. Sig.[a]
Kicking Behaviour-Rearing Behavior	-,013	,316	-,040	,968	1,000
Kicking Behaviour-Bucking Behaviour	-,021	,316	-,066	,947	1,000
Kicking Behaviour-Pulling on the Reins Behaviour	,133	,316	,422	,673	1,000
Kicking Behaviour-Hyperflexion of the Neck Behaviour	,279	,316	,883	,377	1,000
Kicking Behaviour-Desobediences	,296	,316	,936	,350	1,000
Kicking Behaviour-Tail Swishing Behaviour	1,829	,316	5,784	<,001	,000
Kicking Behaviour-Head Shaking Behaviuor	4,096	,316	12,952	<,001	,000
Rearing Behavior-Bucking Behaviour	,008	,316	,026	,979	1,000
Rearing Behavior-Pulling on the Reins Behaviour	,121	,316	,382	,702	1,000
Rearing Behavior-Hyperflexion of the Neck Behaviour	,267	,316	,843	,399	1,000
Rearing Behavior-Desobediences	,283	,316	,896	,370	1,000
Rearing Behavior-Tail Swishing Behaviour	1,817	,316	5,745	<,001	,000
Rearing Behavior-Head Shaking Behaviuor	4,083	,316	12,913	<,001	,000
Bucking Behaviour-Pulling on the Reins Behaviour	,113	,316	,356	,722	1,000
Bucking Behaviour-Hyperflexion of the Neck Behaviour	,258	,316	,817	,414	1,000
Bucking Behaviour-Desobediences	,275	,316	,870	,385	1,000

Pairwise Comparisons

Sample 1-Sample 2	Test Statistic	Std. Error	Std. Test Statistic	Sig.	Adj. Sig.[a]
Bucking Behaviour-Tail Swishing Behaviour	1,808	,316	5,718	<,001	,000
Bucking Behaviour-Head Shaking Behaviuor	4,075	,316	12,886	<,001	,000
Pulling on the Reins Behaviour-Hyperflexion of the Neck Behaviour	,146	,316	,461	,645	1,000
Pulling on the Reins Behaviour-Desobediences	-,163	,316	-,514	,607	1,000
Pulling on the Reins Behaviour-Tail Swishing Behaviour	1,696	,316	5,363	<,001	,000
Pulling on the Reins Behaviour-Head Shaking Behaviuor	3,963	,316	12,531	<,001	,000
Hyperflexion of the Neck Behaviour-Desobediences	-,017	,316	-,053	,958	1,000
Hyperflexion of the Neck Behaviour-Tail Swishing Behaviour	1,550	,316	4,902	<,001	,000
Hyperflexion of the Neck Behaviour-Head Shaking Behaviuor	3,817	,316	12,069	<,001	,000
Desobediences-Tail Swishing Behaviour	1,533	,316	4,849	<,001	,000
Desobediences-Head Shaking Behaviuor	3,800	,316	12,017	<,001	,000
Tail Swishing Behaviour-Head Shaking Behaviuor	2,267	,316	7,168	<,001	,000

Each row tests the null hypothesis that the Sample 1 and Sample 2 distributions are the same.
Asymptotic significances (2-sided tests) are displayed. The significance level is ,050.

a. Significance values have been adjusted by the Bonferroni correction for multiple tests.

Figure S1: Graphical illustration of the pairwise comparisons yielded by by the Related Samples Friedman's Two-Way Test, with Wilcoxon signed-rank test with Bonferroni correction applied post hoc.

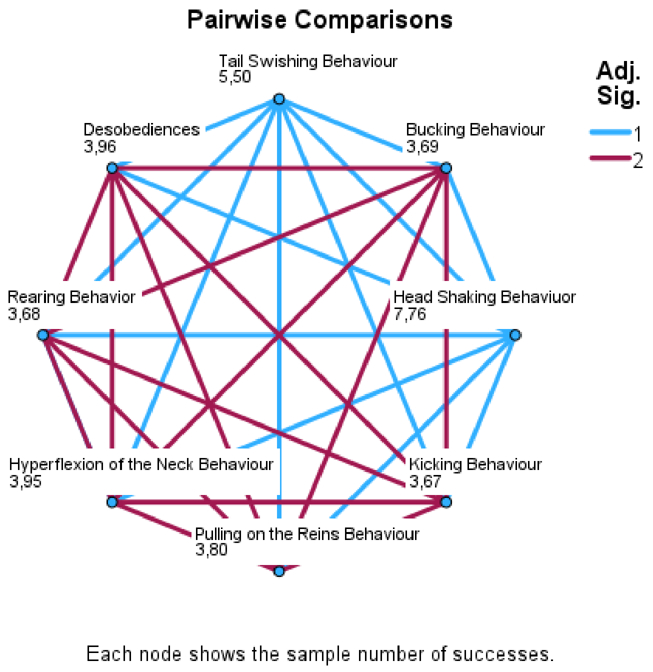

Supplement: Supplementary file 1 [file animals-16-01620-s001.zip › animals-4323286-supplementary.pdf]
